# Coronavirus Outbreak and Stress in Iranians

**DOI:** 10.3390/ijerph17124441

**Published:** 2020-06-20

**Authors:** Masoomeh Maarefvand, Samaneh Hosseinzadeh, Ozra Farmani, Atefeh Safarabadi Farahani, Jagdish Khubchandani

**Affiliations:** 1 Department of Social Work, University of Social Welfare and Rehabilitation Sciences, Tehran 1985713834, Iran; 2 Iranian Scientific Association of Social Work, Tehran 1985713834, Iran; n1713388@gmail.com (O.F.); atefeh.s.farahani@gmail.com (A.S.F.); 3 Department of Biostatistics, University of Social Welfare and Rehabilitation Sciences, Tehran 1985713834, Iran; hosseinzadeh.sam@gmail.com; 4 Department of Nutrition and Health Science, College of Health, Ball State University, Muncie, IN 47306, USA; jkhubchandan@bsu.edu

**Keywords:** coronavirus, pandemic, stress, Iran, outbreak

## Abstract

Iran has faced one of the worst COVID-19 outbreaks in the world, and no studies to date have examined COVID-19-related stress in the general Iranian population. In this first population-based study, a web-based survey was conducted during the peak of the outbreak to assess stress and its correlates in the Iranian population. A 54-item, valid, and reliable questionnaire, including items on demographic characteristics and past medical history, stress levels, awareness about signs and symptoms of COVID-19, knowledge about at-risk groups and prevention methods, knowledge about transmission methods, trust in sources of information, and availability of facemasks and sanitizers, was deployed via social and mass media networks. A total of 3787 Iranians participated in the study where the majority of the participants were females (67.4%), employed (56.1%), from developed provinces (81.6%), without chronic diseases (66.6%), and with ≥13 years of formal education (87.9%). The mean age of study participants was 34.9 years (range = 12–73), and the average stress score was 3.33 (SD = ±1.02). Stress score was significantly higher for females, those who were 30–39 years old, housewives, those with chronic diseases, individuals who were aware that there is no vaccine to prevent COVID-19, those who could not get facemasks or sanitizers, and individuals with higher knowledge about at-risk groups (*p* < 0.05). There was a significant correlation of stress scores with knowledge about prevention methods for COVID-19 (r = 0.21, *p* = 0.01) and trust in sources of information about COVID-19 (r = −0.18, *p* = 0.01). All of the predictors, except knowledge of two important at-risk groups and education, had a significant effect on stress scores based on a multivariate regression model. The COVID-19 outbreak could increase stress among all population groups, with certain groups at higher risk. In the high-risk groups and based on experience with previous pandemics, interventions are needed to prevent long-term psychological effects. Professional support and family-centered programs should be a part of pandemic mitigation-related policymaking and public health practices.

## 1. Background

Since the emergence of the COVID-19 outbreak in China in December 2019, the disease has rapidly spread across the world [1,2,3,4,5,6,7]. By June 2020, COVID-19 had affected more than 8 million people who tested positive, and almost half a million people died worldwide [8]. Iran reported its first confirmed case of COVID-19 infection in February 2020 in Qom [9]. Soon after, other provinces in Iran reported COVID-19 cases, and as a result, schools and universities were closed in the affected provinces, and several cultural, sports, and religious gatherings were canceled as well. In early April 2020, there were 8522 COVID-19-associated deaths worldwide, with a large proportion of deaths being reported from Iran [10]. Travel and other types of warnings and advisories have been issued by the Iranian government regularly since the first case was found [11].

Despite growing attempts to increase public awareness on prevention, people around the world were suffering from widespread fear, stress, and anxiety. This could be more prominent in areas of peak transmission and spread, with people feeling stressed and anxious about the transmission of the disease. For example, a major psychological burden on the public was identified during the peak of the COVID-19 outbreak in China [12,13,14]. Young people, people who spent too much time searching for information or working on the frontlines, healthcare workers with exposure to confirmed or suspected cases, and survivors of COVID-19 had the highest levels of anxiety, depression, and mental distress [13,14,15]. The outbreak itself and the control measures may lead to widespread fear and panic, especially stigmatization and social exclusion of confirmed patients, survivors, and family members, which may escalate into further negative psychological reactions, including adjustment disorder and depression [16].

During the COVID-19 pandemic, Chinese individuals, especially those who were quarantined and had limited access to face-to-face communication, experienced serious psychological problems (e.g., anxiety, psychosis, depression). [16]. In the published literature, post-traumatic stress disorder (PTSD) and depressive disorders have been reported as prevalent long-term psychological consequences of epidemics [13]. Patients with confirmed or suspected COVID-19 may experience fear of the consequences of infection with a potentially fatal new virus, and those in quarantine might experience boredom, loneliness, and anger. For example, in the early phase of the SARS outbreak, a range of psychiatric morbidities, including persistent depression, anxiety, panic attacks, psychomotor excitement, psychotic symptoms, delirium, and even suicidality, was reported [15]. However, much of the evidence on COVID-19 relating to the stress of the pandemic has emerged from China, and few studies have examined the burden of COVID-19-related stress in the general population from other countries. One of the worst COVID-19 outbreaks was reported from Iran, and no studies have examined the stress in the general Iranian population or in the Middle East. Thus, the purpose of this study is to measure Iranians’ stress levels and the associated factors during the COVID-19 outbreak.

## 2. Methods

### Study Participants and Procedures

A web-based cross-sectional study was conducted in the general Iranian population, targeting internet-using volunteers. A multi-item online questionnaire was deployed via the main page of the Iranian Scientific Association of Social Work to the general public. This valid and reliable questionnaire was developed based on a comprehensive literature review and expert panel guidance to measure perceptions and distress during an influenza pandemic [13,14,17,18]. Study participants were recruited using social networks such as Telegram, WhatsApp, and Instagram. This questionnaire was online for 5 days (from 26 February to 1 March 2020), and 3787 Iranians took the survey. The questionnaire could be taken online using a secure HTML interface, where all security conditions for data and personal information were provided to potential study participants. Each questionnaire could be completed only once per device. Respondents were required to answer every question. They were able to review or change their answers before submitting final responses to the questionnaire. Participants were informed about the purpose of the study and emphasized that their participation was voluntary and anonymous. This study was approved by the Iranian Scientific Association of Social Work (98/P/419) for ethical procedures and scientific protocols.

## 3. Measures

A 54-item questionnaire was used to collect data and information in this study. The questionnaire included items about demographic characteristics and past medical history, stress levels, awareness about signs and symptoms of COVID-19, awareness about at-risk groups, knowledge about COVID-19 transmission methods, knowledge about effective COVID-19 prevention methods, awareness of the lack of a vaccine to prevent COVID-19, trust in information sources about COVID-19, and availability of facemasks and sanitizers.

The questionnaire included six questions about demographic characteristics and past medical history to assess gender, age, province of residence, years of formal education, employment status (full-time, part-time, unemployed, housewife, student, or retired), and chronic diseases (including respiratory problems such as asthma and lung disease, cancer, stroke, diabetes, heart diseases such as heart failure and high blood pressure, kidney diseases such as kidney failure, liver diseases such as hepatitis and cirrhosis, psychiatric illnesses such as depression and anxiety, and alcoholism or drug addiction).

Stress-related data was collected by asking five questions about the level of feeling calm, tense, upset, relaxed, and worried. Studies show that participants who receive short questionnaires are more likely to respond [19]. Hence, the expert panel chose a brief set of stress-related questions that have been used in an epidemic situation [17]. For each question, participants could select responses from a set of options (very high, high, moderate, low, and very low, with a score range of 5–1 for each question). The mean of the responses on the five questions measures the stress level of individuals, and a higher score indicates higher stress. Internal consistency reliability of the stress scale was assessed by computing Cronbach’s alpha from the total sample of participants and was found to be high (α=0.81). Additionally, there were five questions about awareness of signs and symptoms of COVID-19 (true or false, with a score range of 1–0 for each question), two questions about the awareness of at-risk groups (true or false, with a score range of 1–0 for each question), four questions about the knowledge about COVID-19 transmission methods (true/false/not sure with a score range of 2–0 for each question), and one statement about the awareness of lack of COVID-19 prevention vaccine: “there is no vaccine for COVID-19” (true/false/not sure with a score range of 2–0). A group of questions (*n* = 19) was included about the knowledge of effective COVID-19 prevention methods (true/false/not sure, with a score range of 2–0 for each question). The internal consistency reliability for this scale (*n* = 19 questions) was assessed by computing Cronbach alpha and was found to be reasonable (α = 0.73).

Ten questions were about the participants’ trust in various sources of information about COVID-19. Sources of information included people they interact with (such as family, friends, and colleagues), health professionals, official websites (such as the Ministry of Health website), health centers (such as hospitals and public health centers), social networks (such as WhatsApp, Telegram, and Instagram), television, radio, newspapers, online news agencies, and international websites such as WHO website. Participants could indicate their level of trust in each of these sources using a 6-point Likert scale (very much, much, moderate, low, very low, and not trustable, with a score range of 5–0 for each question). The mean of the score on the 10 trust-related questions was the average of individual scores of trust in sources of information about COVID-19. To assess the internal consistency reliability of this scale on trust in sources of information, we computed a Cronbach alpha from the final sample of respondents, and the reliability was found to be high (α = 0.83). The questionnaire also included questions about the availability of facemasks and disinfectant gel and sanitizers to assess the individuals’ access to these protective strategies (yes/no, with a score range of 1–0 for each question).

## 4. Data Analysis

Data were in Excel file and were checked for duplicates and any errors before importing and analyzing using IBM SPSS 22 (Chicago, IL, USA). In the primary approach, a descriptive analysis of demographic and background characteristics of study participants was conducted. Next, we compared the average stress scores using *t*-tests or ANOVA based on demographic characteristics, awareness about signs and symptoms of COVID-19, awareness about at-risk groups, knowledge about COVID-19 transmission methods, knowledge about COVID-19 prevention methods, awareness about the unavailability of COVID-19 prevention vaccine, trust in sources of information, and the availability of facemasks and sanitizers. The effect of the predictor variables on stress was assessed by univariate and multivariate generalized linear models. First, each predictor variable was entered into the univariate model separately, then the variables that had *p*-values < 0.2 were entered into the multivariate model simultaneously. Statistical significance was assumed at *p* < 0.05.

## 5. Results

A total of 3787 Iranians with a mean age of 34.9 years (range = 12–73 years) participated in the study. Table 1 illustrates the demographic characteristics of the study population and average stress scores differences among groups. The majority of the study population were females (67.4%), employed (56.1%), from developed provinces (81.6%), without chronic diseases, and had more than 13 or more years of formal education (87.9%). The majority of participants reported that during the last week, they could not get facemasks (74%) or sanitizers and disinfectant gel (50.2%). Less than half (44.5%) of the participants were aware of at least three important symptoms of COVID-19, and most of them (78.5%) knew that elderly people and individuals with background diseases have a higher risk of infection. The majority of participants had very good knowledge about COVID-19 transmission (97%) and prevention (97.3%) methods and knew that there is no approved vaccine for COVID-19 (83.5%). Most of the participants reported that they had high trust in various sources of information about COVID-19 (65.9%). The average scores of participants’ knowledge about the transmission and prevention methods for COVID-19 were 1.72 (±0.28) and 1.72 (±0.27), respectively. The average score on trust in the sources of information about COVID-19 was 2.89 (SD = ±0.85).

The average stress score for the study population was 3.33 (SD = ±1.02). The relationship between demographic characteristics and stress was measured by *t*-test and ANOVA. The mean of stress scores was significantly higher for females, people in the age group of 30–39 years, housewives, those with chronic diseases, individuals who were aware that there is no vaccine to prevent COVID-19, those who could not get facemasks or sanitizers, and individuals who knew about at-risk groups (*p* < 0.05) (Table 1). ANOVA was performed to study the relationship between stress and awareness about symptoms and at-risk groups. The mean of stress scores was statistically significantly different by levels of knowing two important at-risk groups (*p* < 0.05), but there was no significant difference by knowledge on five important symptoms (*p* > 0.05).

Pearson’s correlation coefficients showed that participants’ knowledge about transmission methods of COVID-19 did not correlate with stress scores (r = 0.11, *p* = 0.08), whereas there was a statistically significant correlation of stress scores with knowledge about prevention methods for COVID-19 (r = 0.21, *p* = 0.01) and trust in sources of information about COVID-19 (r = −0.18, *p* = 0.01).

Univariate and multivariate generalized linear models were fitted on the stress scores. Demographic variables (including gender, age, employment, education, province, awareness of no approved vaccine for COVID-19, background disease), knowledge about transmission and prevention methods, awareness about signs and symptoms and at-risk groups, and trust in sources of information about COVID-19 were individually entered in the univariate models. Subsequently, variables that had *p*-values <0.2 were simultaneously entered in the multivariate model (Table 2). All of the variables, except knowledge on the two important at-risk groups and education, were significantly associated with stress scores in the multivariate model.

## 6. Discussion

In this first large national study from Iran, high-levels of stress were reported by the general public during the COVID-19 outbreak. Unfortunately, the long-term effect of such high levels of stress, resulting in serious mental health issues, would be an additional burden on the Iranian public and healthcare system as an aftermath of the pandemic. Our findings provide further evidence of the mental health crises created by emerging infectious disease pandemics. Following the outbreaks of HIV, Ebola, and SARS in other countries, the prevalence of psychological symptoms was reported, and many individuals had experienced long-lasting psychiatric problems [18,20,21,22].

There is an interaction between external conditions (threat) and internal ones (vulnerability) that influences the level of risk for debilitating stress that could lead to mental health problems [20,21,22,23]. In this study, we identified unique groups with high stress. First, across various studies, women have reported high stress. In this study and given the pandemic, it is highly likely that women face multiple and additional stressors, including work, taking care of others in the household, arranging for materials and supplies for the household, and arranging school work and education for children. Their routines have been dramatically changed and profoundly disrupted, causing more stress. Second, individuals with chronic diseases have higher stress in general, and this could be accentuated by the lack of sanitizers, protective masks, and the awareness of lack of a vaccine to prevent COVID-19 infection. The lack of protective supplies and heightened awareness could have severely impacted even those without chronic diseases or lifestyle problems. Third, those in the middle age groups and working-class could have more stress due to multiple social, economic, and personal stressors. It could be possible that they are worried about losing their jobs or their income. Those who had part-time jobs were more stressed than those who had full-time jobs. The instability of part-time jobs, low incomes, and lack of savings could affect some groups more than others. Additionally, healthcare access, coverage, and the ability to pay are influenced by employment, and this could be a major stressor for middle-aged working-class individuals. Fourth, there is an interesting relationship between education and stress levels. Individuals with lower stress had the lowest and highest education levels compared to those who had 13–16 years of formal education. One possible reason could be that during the COVID-19 outbreak in Iran, there was a lot of misinformation circulating on social media networks. Those with lesser education were not generally able to read the information in other languages (such as English) and had lesser access to mass media and technology to use social media. However, with increasing education, this stress seemed to have been alleviated, possibly due to the ability to screen for and use authentic information [24,25,26,27,28,29].

Similar to many epidemics in the past and the response of various governments, it appears that mental health care is not a priority for governments during such national crises, epidemics, and disasters. In addition, factors such as the rural–urban divide, lack of access to technology and authentic information, income inequality, availability of healthcare or other resources, lack of awareness, and literacy could pose additional burdens and cause psychological distress [13,14,15,16,18,20,21,22]. The HIV/AIDS epidemic that captivated world attention in the 1980s and 1990s, the severe acute respiratory syndrome (SARS) in 2002 and 2003, the H1N1 influenza pandemic of 2009, the Ebola virus outbreak in 2013, and the Zika virus outbreak in 2016 are some examples of sudden outbreaks that posed monumental challenges to the mental health service system [18,20,21,22,23]. During these epidemics, the consequences on the psycho-social well-being of at-risk communities were largely overlooked. For example, in the Ebola-affected regions, few measures were taken to address the mental health needs of confirmed patients, their families, medical staff, or the general population, and this resembles the responses to all recent epidemics. The absence of mental health and psycho-social support systems and the lack of well-trained psychiatrists and/or psychologists in these regions increased the risks of psychological distress and progression to psychopathology [21,22,23,24,25].

Continuous surveillance and monitoring of the psychological consequences for outbreaks should become a part of disaster and pandemic preparedness efforts worldwide. Moreover, interventions should be geared towards the most affected and vulnerable individuals as a part of a population mental health promotion strategy [21,22,23,24,25,26,27,28,29,30]. Currently, according to the notification of basic principles for emergency psychological crisis interventions by the National Health Commission of China, mental health care should be provided for patients with COVID-19, close contacts, suspected cases who are isolated at home, patients in fever clinics, families and friends of affected people, health professionals caring for infected patients, and the public [13,14,15,16]. However, Xiang and colleagues claim that the mental health needs of patients with confirmed COVID-19, patients with suspected infection, quarantined family members, and medical personnel have been poorly handled and believed that the organization and management models for psychological interventions in China must be improved [15]. Similar challenges are now being seen across the severely affected countries and will continue to emerge in regions that will be severely affected with COVID-19 or any future pandemics and epidemics [23,24,25]. Social media, fear and misinformation, myths, and rumors have added to the global burden of information overload and psychological distress [26,27,28]. Pandemic response policies should include mental health promotion practices as a key initiative in dealing with the psycho-social burden of pandemics. Additional research is needed in the form of long-term prospective studies to assess the causal mechanisms and impact of stress caused by the COVID-19 pandemic.

## 7. Limitations

The results of this study are subject to several potential limitations. First, the study results are restricted by all traditional limitations of cross-sectional study designs (e.g., reliance on self-reported behaviors, recall bias in participants, socially desirable responses, and the inability to establish cause-and-effect relationships). Second, this study measured the prevalence of variables rather than incident cases, and we were unable to assess the levels of variables before COVID-19 outbreak in Iran as a control. Third, stress patterns in individuals often have a variety of simultaneous influences that need to be accounted for. Fourth, a major threat to external validity is that the sample is limited in nature and extent (e.g., dominated by female participants, those who were employed and <49 years old, and from developed provinces). This would mean that the results of the study cannot be generalized to several groups of individuals across the region and other countries. Despite these limitations, our study is the first and largest study across the middle East, with robust measures, of COVID-19-associated stress and the factors associated with stress during the pandemic.

## 8. Implications for Practice

Based on our field experiences and a comprehensive review of literature, we recommend the following strategies for mental health promotion and disease mitigation in communities during the COVID-19 pandemic or any future epidemics or disasters [24,25,26,27,28,29,30,31,32,33,34].

People may speculate and/or spread rumors, myths, and misinformation about COVID-19 or infectious disease agents. This has been a major problem with the current pandemic, given the widespread use of mass and social media methods, increasing stress and spreading panic. It is essential to encourage the public not to spread misinformation and inform them of verified and credible sources of information. Governments should engage in effective and efficient risk communication and share information regarding disease prevention methods and strategies.

Governments and scientific organizations should provide clear information about COVID-19 or any infectious disease-related symptoms, signs, transmission methods, and prevention strategies through scientific websites, daily briefings, and public awareness campaigns. This will help citizens deal with pandemics more effectively and also mitigate the chain of transmission and spread of disease. Denying epidemics and the associated population health risks will cause a loss of confidence in the government and credibility of scientific organizations, which poses an additional burden on disease management and population health services. Lack of effective flow of information and disease-related data and statistics has been a major problem worldwide with the COVID-19 pandemic.

People may be worried, anxious, and depressed due to the constantly changing alerts, media cycle, and social or mass media coverage regarding the spread of COVID-19. Therefore, providing psychological support services by volunteer social workers, psychologists, psychiatrists, and counselors could help alleviate stress and persistent excessive arousal. In this regard, long-distance psycho-social support (including telecounseling and online services) can be deployed. Because people’s routines may change dramatically during pandemics and quarantine periods, they should receive compassionate services and guidance on becoming familiar with alternative programs and lifestyles. Opening online platforms with audio- and video-based information and demonstrations and providing counseling services and telephone helplines for those with new-onset symptoms or existing mental illnesses can be used until routine mental healthcare services are available.

Grief counseling for people who have lost their family members due to COVID-19, or those who lose family members due to other epidemics and disasters, is critical. Strategies such as stress reduction, conflict resolution, crisis call centers, child protection, and custody conflict mitigation are some areas of emphasis for psycho-social service providers. It is also so important to pay attention to the possibility of deprived families who have infected members due to the stigma caused by the disease, and provide special support for them to ensure their access to essential facilities and services.

While our study did not specifically look at frontline workers, and essential service and healthcare professionals, existing evidence indicates the heavy toll that pandemics such as COVID-19 can take on the physical and mental health of certain at-risk groups. Disrupted sleep–wake cycle, patient and client overflow, long working hours, changing practice protocols, fractured communication, and shortage of materials, equipment, and supplies can cause high stress among these populations in disasters and pandemics. Individual-level, intrapersonal, and organizational level interventions to reduce stress and burnout should be implemented (e.g., shift rotations, work hours limitation, employee assistance programs, enforcing protocols, coordinated workflow and information processes, just to name a few).

A major stressor among individuals and families during epidemics is financial or economic. Governments should support employers and strengthen social protection, especially for vulnerable families (including elder people, disabled or sick people, and female-headed households). Family-friendly policies and programs are necessary to support affected people during the COVID-19 pandemic (including employment and income protection, flexible working arrangements, paid leave to care for family members and access to health care and medical services, direct benefit transfers, food distribution for needy families, among others). Governments across the world have rolled out stimulus plans and advisories for tax and debt moratoriums that should provide temporary assistance to needy families.

Voluntary activities during this period can significantly contribute to social solidarity and social cohesion. It is recommended that these activities be encouraged. Skilled volunteer professionals could be involved in a wide range of activities to reduce the economic, social, and health impacts of the epidemic. Nonprofit organizations can play a key role in mobilizing skilled volunteer professionals. The no-harm principle is essential to providing volunteer services during an epidemic.

## 9. Conclusions

The COVID-19 outbreak has severely increased psychological distress among people in Iran. It has changed people’s routines in several aspects and made it difficult to cope with the new situation. Affected people, especially vulnerable groups, need professional support and community-based mental health promotion services to deal with the multiple stressors and burden imposed by the current pandemic. Family-centered social and economic policies and programs are necessary to support people during the COVID-19 epidemic. Employment and income protection, flexible working arrangements, paid leave to care for family members and access to medical services, cash transfers, and food distribution for low-income or no-income families, and providing long-distance psycho-social interventions are some examples of family-friendly policies to support people during this pandemic or any future epidemics of a regional or global nature.

## Figures and Tables

**Table 1:** Demographic characteristics of the participants and the mean of stress scores in groups.

Variables	Categories	N	%	Stress Score (M ± SD)	Test Value	*p*-Value
Gender	Female	2553	67.4	3.45 ± 1.02	T = 11.107	<0.001
	Male	1234	32.6	3.07 ± 0.99		
Age (years)	≤29	1145	30.2	3.27 ± 1.05	F = 12.97	<0.001
	30–39	1551	41.0	3.45 ± 1.01		
	40–49	710	18.7	3.23 ± 1.02		
	≥50	381	10.1	3.20 ± 0.96		
Employment	Full-time	1479	39.0	3.32 ± 1.00	F = 10.678	<0.001
	Part-time	646	17.1	3.40 ± 0.99		
	Housewife	486	12.8	3.55 ± 1.01		
	Student	578	15.3	3.29 ± 1.03		
	Others	601	15.9	3.22 ± 1.08		
Education (year)	≤12	458	12.1	3.24 ± 1.04	F = 3.226	0.040
	13–16	1741	46.0	3.37 ± 1.03		
	≥17	1588	41.9	3.31 ± 1.00		
Provinces	Developed	3090	81.6	3.63± 1.02	F = 3.988	0.019
	Developing	287	7.6	3.34 ± 1.07		
	Less developed	410	10.8	3.19 ± 1.02		
Chronic diseases	Yes	1263	33.4	3.51 ± 1.02	T = 7.936	<0.001
	No	2524	66.6	3.24 ± 1.01		
Availability of facemask	Yes	598	26.0	3.37 ± 1.01	T = 3.711	<0.001
	No	1701	74.0	3.54 ± 0.97		
Availability of sanitizer and disinfectant gel	Yes	1318	49.8	3.33 ± 1.01	T = 6.107	<0.001
	No	1331	50.2	3.56 ± 0.97		
The number of signs and symptoms that the participants were properly aware of	0	44	1.2	3.26 ± 1.12	F = 1.692	0.133
	1	433	11.4	3.29 ± 1.02		
	2	524	13.8	3.24 ± 1.06		
	3	1695	44.5	3.38 ± 1.01		
	4	825	21.8	3.31 ± 1.00		
	5	266	7.0	3.32 ± 1.06		
The number of at-risk groups that the participants were properly aware of	0	20	0.5	2.92 ± 1.16	F = 6.393	0.002
	1	787	20.8	3.23 ± 1.02		
	2	2980	78.7	3.36 ± 1.02		
Knowledge about COVID-19 transmission methods	Low (<1)	114	3	3.19 ± 1.08	T = 1.49	0.136
	High (≥1)	3673	97	3.33 ± 1.02		
Knowledge about COVID-19 prevention methods	Low (<1)	103	2.7	2.57 ± 1.11	T = 7.67	<0.001
	High (≥1)	3684	97.3	3.35 ± 1.01		
Awareness of lack of vaccines for COVID-19 prevention	Yes	3164	83.5	3.36 ± 1.02	T = 4.360	<0.001
	No	623	16.5	3.17 ± 1.05		
Trust in information sources about COVID-19	Low (<2.5)	1288	34.1	3.47 ± 1.06	T = 6.30	<0.001
	High (≥2.5)	2499	65.9	3.25 ± 0.99		

M ± SD = mean stress scores compared among groups in each variable (range = 5–25). Test statistics = values of statistical tests such as *t*-tests (t) and ANOVA (F).

**Table 2:** Predictors of COVID-19-related stress in the general Iranian public.

Variables (ref.)	Levels	Univariate Models			Multivariate Model		
		B	SE	*p*-Value	B	SE	*p*-Value
Knowledge about COVID-19 transmission methods		0.179	0.088	0.042	0.129	0.057	0.024
Awareness of COVID-19 prevention methods		0.401	0.183	<0.001	0.764	0.061	<0.001
Trust in sources of information about COVID-19		−0.094	0.028	0.001	−0.146	0.019	<0.001
Number of known important symptoms		0.016	0.048	0.307			
Number of known important at-risk groups		0.136	0.039	<0.001	0.072	0.037	0.052
Age (≤29)	30–39	0.175	0.040	<0.001	0.065	0.043	0.134
	40–49	−0.049	0.049	0.317	−0.183	0.052	<0.001
	≥50	−0.078	0.060	0.193	−0.239	0.063	<0.001
Gender (male)	Female	0.384	0.035	<0.001	0.276	0.037	<0.001
Education (Year) (≤12)	13–16	0.129	0.054	0.016	0.040	0.052	0.436
	≥17	0.074	0.054	0.172	−0.020	0.055	0.718
Employment (Full-time)	Part-time	0.085	0.048	0.076	−0.037	0.047	0.429
	Housewife	0.231	0.053	<0.001	−0.002	0.055	0.974
	Student	−0.120	0.050	0.016	−0.209	0.056	<0.001
	Others	−0.097	0.049	<0.001	−0.168	0.048	<0.001
Provinces (Developed)	Developing	0.010	0.063	0.994	0.040	0.060	0.507
	Less developed	−0.151	0.054	0.005	−0.114	0.052	0.027
Chronic disease (No)	Yes	0.278	0.035	<0.001	0.296	0.034	<0.001
Awareness of no approved vaccine for COVID-19 (No)	Yes	0.195	0.045	<0.001	0.179	0.043	<0.001
